# Increased Sleep Fragmentation Leads to Impaired Off-Line Consolidation of Motor Memories in Humans

**DOI:** 10.1371/journal.pone.0034106

**Published:** 2012-03-28

**Authors:** Ina Djonlagic, Julian Saboisky, Andrea Carusona, Robert Stickgold, Atul Malhotra

**Affiliations:** 1 Division of Sleep Medicine, Brigham and Women's Hospital, Boston, Massachusetts, United States of America; 2 Department of Psychiatry, Beth Israel Deaconess Medical Center, Boston, Massachusetts, United States of America; 3 Harvard Medical School, Boston, Massachusetts, United States of America; University of Pennsylvania School of Medicine, United States of America

## Abstract

A growing literature supports a role for sleep after training in long-term memory consolidation and enhancement. Consequently, interrupted sleep should result in cognitive deficits. Recent evidence from an animal study indeed showed that optimal memory consolidation during sleep requires a certain amount of uninterrupted sleep.

Sleep continuity is disrupted in various medical disorders. We compared performance on a motor sequence learning task (MST) in relatively young subjects with obstructive sleep apnea (n = 16; apnea-hypopnea index 17.1±2.6/h [SEM]) to a carefully matched control group (n = 15, apnea-hypopnea index 3.7±0.4/h, p<0.001. Apart from AHI, oxygen nadir and arousal index, there were no significant differences between groups in total sleep time, sleep efficiency and sleep architecture as well as subjective measures of sleepiness based on standard questionnaires. In addition performance on the psychomotor vigilance task (reaction time and lapses), which is highly sensitive to sleep deprivation showed no differences as well as initial learning performance during the training phase. However there was a significant difference in the primary outcome of immediate overnight improvement on the MST between the two groups (controls = 14.7±4%, patients = 1.1±3.6%; *P* = 0.023) as well as plateau performance (controls = 24.0±5.3%, patients = 10.1±2.0%; *P* = 0.017) and this difference was predicted by the arousal index (p = 0.02) rather than oxygen saturation (nadir and time below 90% saturation. Taken together, this outcome provides evidence that there is a clear minimum requirement of sleep continuity in humans to ensure optimal sleep dependent memory processes. It also provides important new information about the cognitive impact of obstructive sleep apnea and challenges its current definitions.

## Introduction

Over the last few decades, advances in cognitive neuroscience have expanded our understanding of the functional and neural composition of memory systems.

Improvement of a skill can be enhanced by ongoing training, but it can even continue afterwards through off-line processes [1]. Sleep is a complex state that has been shown to promote off-line memory consolidation and its underlying plastic processes. Only over the past decade has sleep become recognized as a state favorable for brain plasticity [2], [3], [4].

An established paradigm to study sleep-dependent consolidation of non-declarative procedural memory has been the motor sequence task (MST). Motor skill learning is a fundamental human activity that supports the effortless performance of activities ranging from tying a shoe to playing a Chopin piano sonata through repeated practice [5]. It is one component of the non-declarative memory system, involving several brain structures, including the neocortex, neostriatum and cerebellum [6].

Motor memories improve during off-line periods with sleep being of particular importance. Studies looking at different aspects of motor skill learning, including motor sequence, and motor adaptation tasks, have shown improvements in performance after a night of sleep, but not after an equivalent time spent awake [7], [8]. For example, results from the MST have shown absolute improvement by 20% in performance over a period of 24 h in the absence of additional training [9]. Across a 12 hour training-retest interval, this improvement was seen only when the interval contained a night of sleep, and not after 12 hours of wake [10]. Functional imaging studies suggest that sleep facilitates the systems level reorganization of the memory, resulting in functional neural changes that lead to enhanced performance of the newly learned material [10].

Compelling evidence for not only sleep per se, but also continuous periods of sleep being of significance for optimal memory consolidation came from a recently published animal study manipulating sleep continuity in mice with optogenetics. This study showed that sleep fragmentation following the acquisition of a novel object recognition task led to reduced performance compared to a control group that was allowed to sleep naturally. By selectively modifying sleep continuity without altering duration, composition and intensity, the authors showed that as microarousals increase, subsequent memory performance decreases [11].

To our knowledge there have been no previous studies in humans, looking precisely at the effect of sleep fragmentation caused by microarousals without altering the sleep architecture on sleep-dependent memory consolidation.

Obstructive sleep apnea (OSA) is a common sleep disorder leading to sleep fragmentation and intermittent hypoxia. Previous research has demonstrated the various levels of cognitive deficits in patients with sleep apnea, which extend beyond those primarily associated with sleepiness. In particular, because mild obstructive sleep apnea is characterized by frequent arousals without significant oxygen desaturations it represents a suitable human model to investigate the effects of such fragmentation on memory consolidation.

We tested directly the effect of mircoarousals on motor memory consolidation by comparing a group of young patients with OSA to a carefully matched control group in order to address the following 2 hypothesis:

1. When compared to healthy controls, patients with mild OSA and a higher arousal index will show only practice-related learning (encoding) and not off-line sleep-dependent enhancement.
2. This observed deficit is independent of circadian factors as well as differences in attention and vigilance.

**Figure 1:**
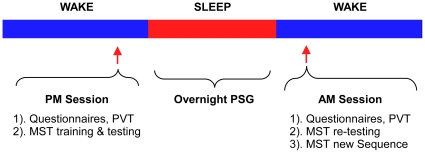
Schematic overview of the protocol. All subjects were trained in the evening between 8 and 9 PM and re-tested the following morning between 6:30 and 7:30 AM on the MST. After testing, all subjects learned a new MST sequence in the morning to control for circadian effects of learning. The PVT was applied before evening and morning sessions to control for potential differences in attention and vigilance between both groups.

We studied 16 patients with mild OSA and 15 controls and compared their training performance on 12 trials of the motor sequence learning task in the evening to performance on 12 test trials in the morning following a full night of sleep measured by polysomnography (see Figure 1). In order to control for vigilance states, all subjects performed the psychomotor vigilance task (PVT) in the evening and in the morning before the MST. Learning of the MST is sequence specific with no transference of learning to new sequences [12]. This finding allowed us to control for a circadian effect in motor sequence learning by having all subjects learn a new sequence of the MST in the morning following the testing of the sequence from the previous evening. All subjects also completed standard sleepiness scales to measure subjective levels of sleepiness.

## Results

### Demographic and PSG data

Subjects were recruited from people, who were referred to the sleep lab for an overnight sleep study and then assigned to be either in the OSA group (AHI>5/h) or the control group. OSA patients (n = 16) and controls (n = 15) were similar in age and BMI (Table 1). Analysis of polysomnograms demonstrated no significant difference in total sleep time, sleep efficiency or sleep stage distribution between groups. The only significant differences were seen in AHI (17.1±2.6/h vs. 3.7±0.4/h, p<0.001), oxygen nadir (87.4±1.1% vs. 91.1±0.7%, p = 0.004) and arousal index (25.0±3.0/h vs. 16.7±1.2/h, p = 0.020; Table 1)

**Table 1:** Demographic and Sleep parameters.

	OSA (n = 16)	Controls (n = 15)	*p* Value	*R^2^* Value
**Age (years)**	31.9±1.7	29.0±1.7	0.48	0.04
**BMI (Kg/m^2^)**	29.7±1.9	27.9±1.53	0.24	0.02
**ESS**	9.5±1.1	9.9±1.6	0.81	0.09
**SSS (evening)**	3.0±0.3	3.7±0.4	0.19	0.07
**SSS (morning)**	3.3±0.3	3.1±0.4	0.68	0.11
**PSG data**				
**Total sleep time (min)**	319.3±14.1	345.0±5.8	0.11	0.02
**Sleep efficiency (%)**	82.4±2.8	85.5±2.8	0.45	0.04
**Stage N1 (%)**	7.7±1.1	8.8±1.5	0.55	0.02
**Stage N2 (%)**	63.8±1.9	58.6±2.3	0.10	0.00
**Stage N3 (%)**	13.3±2.5	14.9±3.3	0.70	0.03
**REM (%)**	15.2±1.8	17.6±2.1	0.39	0.04
**AHI (events/hr)**	17.1±2.6	3.7±0.4	<0.001	0.13*
**REM-AHI**	20.9±4.2	7.0±1.2	0.007	0.03
**4% Oxygen Desaturation Index**	8.9±2.2	1.6±0.4	0.004	0.06
**Oxygen Nadir (%)**	87.4±1.1	91.1±0.7	0.004	0.07
**Time below 90%**	3.25±1.86	0.13±0.09	0.12	0.03
**Arousal Index (events/hr)**	25.0±3.0	16.7±1.2	0.02	0.20*

Definition of abbreviations: BMI = body mass index, AHI = apnea hypopnea index, ESS = Epworth Sleepiness, Scale, SSS = Stanford Sleepiness Scale.

Data are presented as mean ± SEM.

R^2^ value refers to correlation between overnight improvement and parameter (* indicates a significant difference, p≤0.05).

### MST results

#### Practice-dependent improvement

**Figure 2:**
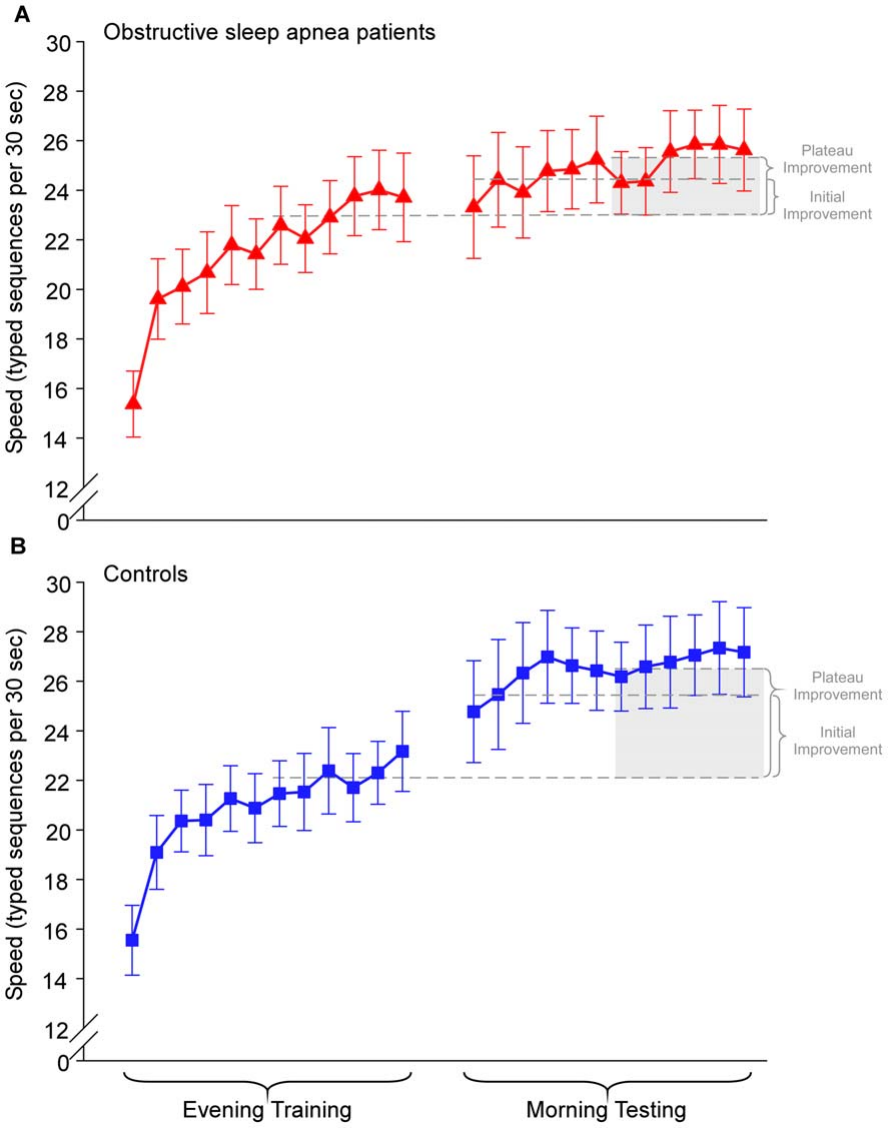
Trial-by-trial performance during evening training and morning testing. Improvement in performance speed on the motor sequence task (MST) across initial training (12 trials) in the evening and at morning re-testing (12 trials) for healthy controls (n = 15, blue squares) and OSA subjects (n = 16, red triangles). The dashed line represents the average performance of the last 6 trials during the evening training as a reflection of the amount of training-dependent learning. Patients and controls did not differ in training-dependent learning in the evening. Overnight change in performance was calculated as *initial improvement* = percent increase of improvement from the last three training trials in the evening to the first three test trials in the morning and *plateau improvement* = percent improvement from the last 6 training trials in the evening to the last 6 test trials in the morning. OSA patients had a plateau improvement of 2.1±0.3 seq/30 sec compared to controls, who showed 5.0±0.9 seq/30 sec (p = 0.003). For the immediate improvement, OSA patients showed an improvement of 0.4±0.8 seq/30 sec compared to controls, who improved by 3.4±1.0 seq/30 sec (p = 0.022). Error bars represent standard errors of the mean (SEM).

We calculated improvement during the evening training session as the increase in correctly typed sequences from the first trial to the average of the last three trials. Both groups showed similar learning curves during training, with the OSA patients even performing slightly better. (Figure 2)

#### Overnight improvement

We used two different measures to assess overnight changes in performance: *initial improvement* = percent increase of improvement from the last three training trials in the evening to the first three test trials in the morning and *plateau improvement* = percent improvement from the last 6 training trials in the evening to the last 6 test trials in the morning. In addition, we examined the *initial lag*, which reflects the delay in expressing the plateau level of improvement and was calculated as plateau improvement minus initial improvement.

**Figure 3:**
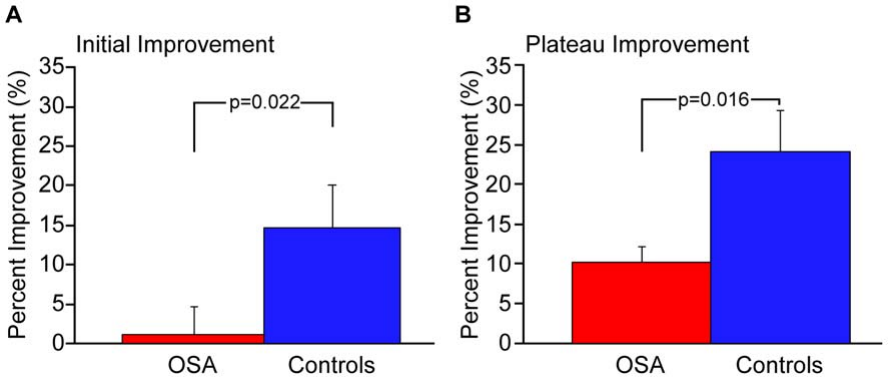
Measurements of overnight performance changes. Immediate and Plateau improvement of OSA patients and healthy controls on the motor sequence learning task (MST). Performance is measured as correctly typed sequences per 30-second trial. There was a difference in off-line improvement over a night of sleep with the healthy controls showing significantly more initial improvement (*P* = 0.023) and plateau improvement (*P* = 0.017). Error bars represent standard errors of the mean (SEM).

The control group showed significantly more immediate overnight improvement (14.7±4.4%) than the OSA patients (1.1±3.6%) p = 0.023; Figure 3, left). Importantly, this effect was not due to a difference in initial lag at the beginning of the morning test session, which reflects a delay in reaching the plateau level from the average of the first three trials. OSA patients showed an initial lag of 9.1±4.5%, which was similar in controls: 8.1±3.2% (p = 0.852).

Learning the MST over 12 trials typically follows the course of an initially steeper increase in performance, which then plateaus towards the end of the session. This plateau can then be analyzed from session to session by comparing the percent improvement from the last six training trials in the evening to the last six test trials the following morning. Similar to the initial improvement, controls show a significantly larger overnight improvement of plateau performance (controls = 24.0±5.3% vs. OSA = 10.1±2.0%; p = 0.017; see Figure 3)

**Figure 4:**
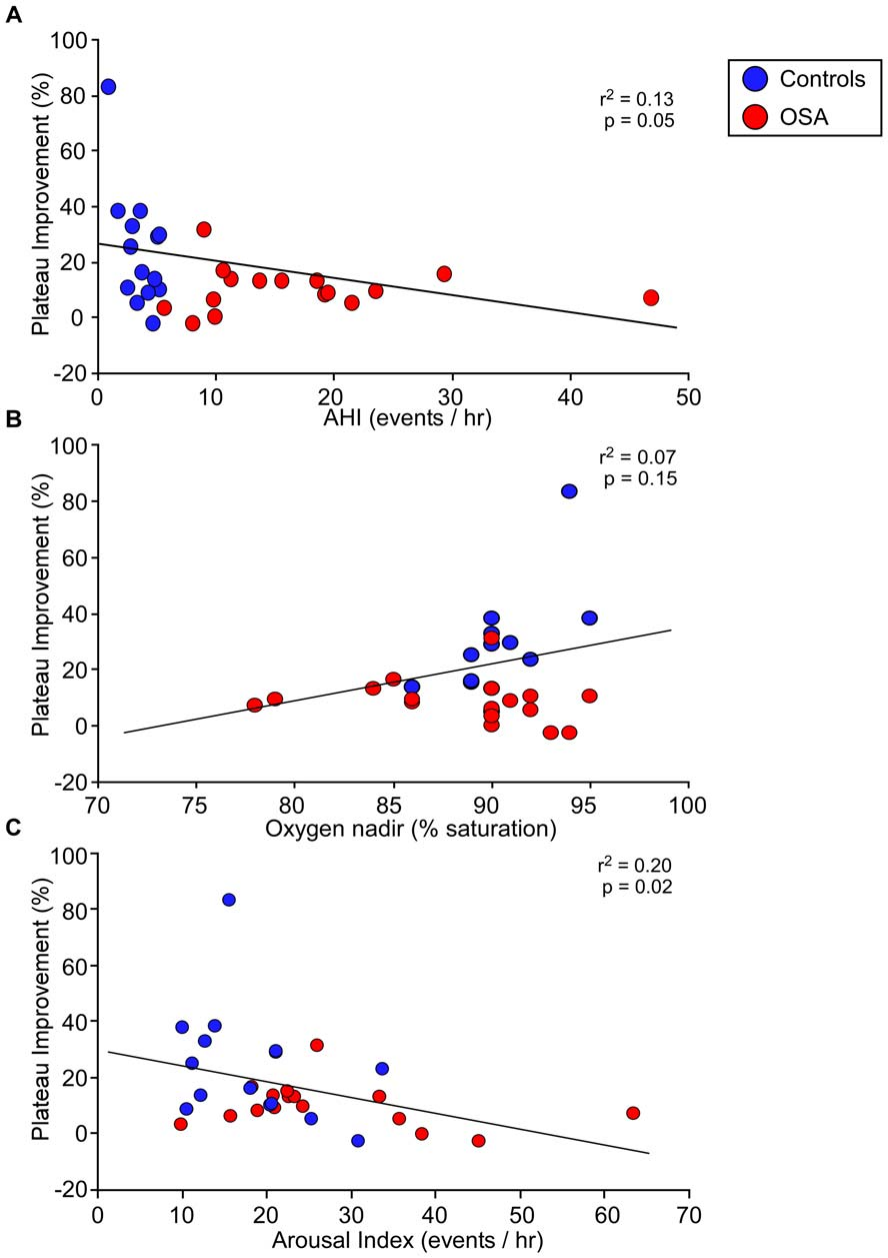
Correlation analysis. Correlations between overnight improvement and AHI (events/hr), oxygen nadir (%) and arousal index for healthy controls (blue circles) and OSA subjects (red circles). Significant correlations were found between overnight improvement and arousal index (r^2^ = 0.20, *P* = 0.02), and, to a lesser extent, between overnight improvement and AHI (r^2^ = 0.13, P = 0.05). In contrast, no significant correlation was seen with oxygen nadir (r^2^ = 0.07, *P* = ns). One healthy control had an overnight plateau improvement of 83%. This individual had a plateau performance of 15 seq/30 sec during evening training (control average = 22 seq/30 sec), which increased to a plateau average of 28.4 seq/30 sec during morning testing (control average = 27 seq/30 sec), thus remaining within the range of normal in regards to the absolute values. Even though this person's percent overnight improvement was well above the average, the correlations remain significant when this individual is removed.

Subjects were randomized to one of two sequences in the evening: 4-2-3-1-4 (sequence A) or 2-4-1-3-2 (sequence B). After testing on the same sequence the following morning, all subjects were trained on the alternate sequence. As displayed in Figure 4, there were no significant differences within groups between performance of the new sequence learned in the morning and initial performance in the evening, ruling out circadian influences as the source of overnight changes. As with the initial training session in the evening, there were no group differences between OSA and controls when learning a new sequence in the morning, which additionally rules out any circadian difference in performance when learning the MST.

### Assessment of sleepiness and alertness

#### By questionnaires

General sleep propensity was assessed with the Epworth Sleepiness Scale (ESS). There were no significant group differences, with both groups averaging in the high normal range (9.5±1.1 vs. 9.9±1.6, p = 0.81).

Similarly, there were no significant group differences in the subjective sleepiness, measured prior to each session with the seven-point Stanford Sleepiness Scale (SSS) between training and re-test. Mean values for OSA patients and controls were 3.0±0.3 vs. 3.7±0.4 (p = 0.19) in the evening and 3.3±0.3 vs. 3.1±0.4 (p = 0.68) in the morning. (Table 1)

#### By Psychomotor Vigilance Test (PVT)

In the evenings, mean reaction times for the PVT were 387.3±34.8 ms (lapses [RT>500 ms] = 8.0±2.8) for OSA subjects and 412±31.9 ms (lapses = 11.5±3.6) for controls. Correspondingly, in the mornings, mean reaction times were 437.1±43.6 ms (lapses 10.1±3.4) for OSA subjects and 419.5±29.9 ms (lapses 11.5±3.2) for controls.

Within subject comparison showed no significant difference between the evening and the morning sessions for the OSA group (p = 0.337 [mean RT], p = 0.642 [lapses]) or for the controls (p = 0.869 [mean RT], p = 0.989 [lapses]). In addition, there was no significant difference between groups for performance during the evening session (p = 0.615 [mean RT], p = 0.440 [lapses] or the morning session (p = 0.717 [mean RT], p = 0.766 [lapses]).

Taken together, these findings indicate an absence of significant differences in sleepiness or vigilance either between groups or between test times.

### Correlation with sleep parameters

**Figure 5:**
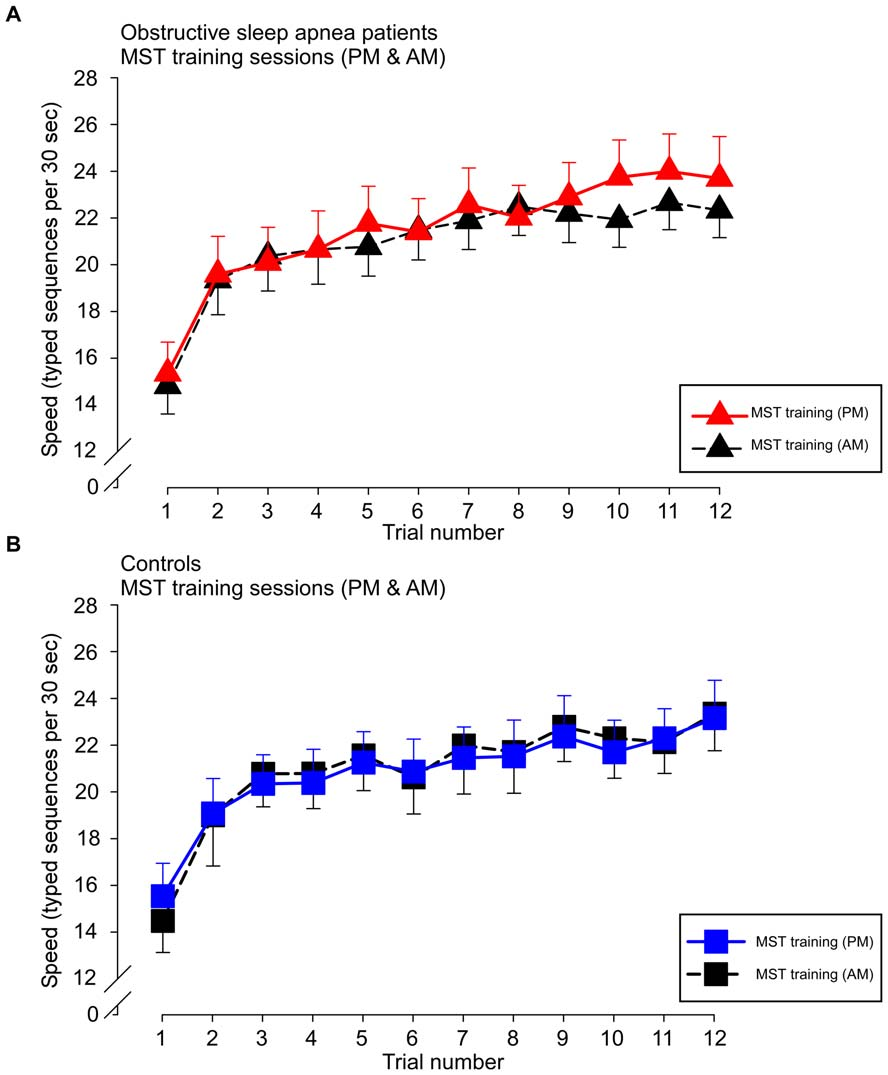
Training performance in the evening compared to morning. Both, OSA patients and healthy controls, showed similar performances during their initial training session in the evening compared to training of a new sequence in the morning. Subjects were randomized to one of two sequences in the evening: 4-2-3-1-4 (sequence A) or 2-4-1-3-2 (sequence B). After testing on the same sequence the following morning, all subjects were trained on the alternate sequence. There were no significant differences within groups (healthy controls or OSA patients) between performance of the new sequence learned in the morning and initial performance in the evening, ruling out circadian influences as the source of overnight changes. Error bars represent standard errors of the mean (SEM).

Regression analyses revealed no significant correlations with any sleep stages (Table 1). However, significant correlations were found between overnight improvement and arousal index (r^2^ = 0.20, p = 0.02), and, to a lesser extent, between overnight improvement and AHI (r^2^ = 0.13, p = 0.05). On the other hand, no significant correlation was found between overnight improvement and REM-AHI (r^2^ = 0.06, p = 0.19) and NREM-AHI (r^2^ = 0.08, p = 0.11) In addition, no significant correlation was seen with oxygen nadir (r^2^ = 0.07, p = 0.13) (Figure 5). This was also the case for the 4% oxygen desaturation index (r^2^ = 0.06, p = 0.16) and time below 90% saturation (r^2^ = 0.03, p = 0.43).

## Discussion

Our study results demonstrate that sleep fragmentation, as induced by OSA can affect off-line learning improvement on a motor sequence learning task and thus highlight the importance of minimizing arousals from sleep for optimal memory consolidation.

In this study, OSA patients and controls were very well matched for standard demographic parameters (age, BMI), subjective assessment of sleepiness (ESS, SSS) and did not show alterations for sleep architecture and duration (TST, sleep efficiency, as well as percent and absolute % time in N1, N2, N3, and REM). This close matching allowed us to separate the consequences of hypoxemia versus arousals on overnight memory consolidation. We were able to distinguish these two effects by showing correlations with overnight improvement only for the arousal index and, to a lesser extent for the total AHI, but not for oxygen measures including oxygen nadir, 4% desaturation index and time below 90% saturation. These results would also explain why earlier studies looking only at AHI or apnea-related arousals failed to show relationships between memory impairments and disease severity [13], [14], [15].

However, arousals per se are obviously not unfavorable. Current data have supported the evolutionary perspective that arousals from NREM sleep comprise a dynamic system, which connects the sleeping person with the surrounding world in order to adapt to possible dangers and guarantee the reversibility of sleep, without which it would be identical to coma [16], [17]. Thus, while physiological arousals are considered a characteristic of stable sleep, those that have been externally induced by experimental conditions or disease such as in OSA can have detrimental effects on off-line plastic processes during sleep.

Keeping in mind that memory is not a unitary process, our study design enabled us to specify further the stages at which memory processes are affected. Independent of the type of memory, deficits in memory performance can occur principally at three levels: encoding, consolidation and retrieval. Each level involves specific brain regions and, to some extent, specific brain states (*e.g.*, wake or sleep), and each of which has its own functional implications. Encoding refers to the initial process during which new information is acquired and “stored” within neural networks. Consolidation includes distinct processes during which initially labile information is stabilized, through both synaptic level and systems level restructuring, into long-term storage. Finally, retrieval is the process by which stored information is accessed, either for conscious recall or to inform behavior.

Successful encoding can be affected by prior sleep deprivation and even mild sleep disruption through changes in hippocampal activation. Therefore, tasks that mainly rely on these structures for optimal processing show impairments, whereas those that do not seem relatively resilient [18], [19].

Given that the MST is a procedural task which does not seem to rely as much on hippocampal structures, it is perhaps not surprising that our OSA patients and controls showed almost identical performance during the initial learning session in the evening, suggesting that there is no difference in encoding of the newly learned information. Our mild OSA patients even averaged slightly higher at the end of training than the control group (23.2 vs. 22.2 seq/30 s, see Figure 2). Both groups established the same level of performance during encoding when they learned a novel MST sequence in the morning, which ruled out a circadian effect on the encoding processes as well as testing performance after a night of sleep.

To address the possibility that differences in attention and vigilance could act as potential confounders on performance during training and re-testing, all participants performed a 5-minute version of the PVT prior to each MST session as an objective measure of behavioral alertness. The lack of significant difference in PVT speed and lapses - the latter being a sensitive marker of diurnal impairment in patients with sleep-disordered breathing - between evening and morning sessions, or between groups, suggests further that alterations specifically in sleep-dependent memory processes must be a source of the deficit in overnight improvement in patients with obstructive sleep apnea. Similarly, OSA subjects and controls did not differ in subjective measures of sleepiness as assessed by the Epworth Sleepiness or Stanford Sleepiness Scales. While both groups scored relatively high on these scales, only the controls exhibited the normal overnight improvement in MST performance, while OSA patients did not. Control subjects, though without clinically meaningful sleep-related breathing problems, had all been referred for an overnight sleep study. Consequently, there was some referral bias, which is most likely reflected in their relatively high ESS scores (compared to the community). Indeed, their post hoc assignment to the control group reflects their being at the lower end of a continuum, rather than being from a discrete population. Nevertheless, these control subjects showed an overnight improvement similar to that reported for healthy college students [10]. This finding further underlines the potential lack of correlation between subjective measures of sleepiness and actual performance and excludes these scales from serving as a predictor for successful overnight memory consolidation [20].

In conclusion, concurrent with recent animal research, we suggest that increased arousals from sleep constitute an important predictor of sleep dependent memory processes presumably interrupting the transfer of labile memories from the hippocampus to the neocortex for long-term storage. The results of our study are also of considerable clinical relevance and raise critical concerns since at the present time, many authorities (*e.g.*, Medicare, AASM recommended criteria) have suggested that arousals from sleep be largely ignored in evaluating OSA patients.

The effects of OSA on off-line plasticity processes during sleep have previously not been well defined. Having a better understanding of the impact of OSA on cognitive processes can help healthcare providers improve diagnostic sensitivity and specificity, and provide appropriate and timely treatment. On the basis of our data, it would be justifiable to minimize arousal from sleep for optimal memory consolidation by treating patients even with mild OSA, thereby challenging current OSA definitions that focus primarily on hypoxemia.

## Materials and Methods

### Ethics Statement

Informed written consent was obtained from all participants and the study was approved by the Brigham and Women's Hospital Institutional Review Board.

### Subjects

We recruited 31 right-handed men and women, between the ages of 18–45 years, who were referred for an overnight polysomnogram (PSG) by their physician.

Subjects were assigned post hoc to either the OSA or healthy control group. Assignment to the OSA group required a new diagnosis of OSA with an apnea-hypopnea index (AHI) of >5/h and no prior exposure to CPAP.

### Subject exclusion criteria

Subjects were excluded if they (1) were found to have a periodic limb movement index of >15/h based on their PSG, (2) had a diagnosed other sleep disorder, (3) had a history of alcohol, narcotic, or other drug abuse, (4) had a history of a medical, neurologic or psychiatric disorder (other than OSA and treated hypertension) that could influence excessive daytime sleepiness, (5) used medications known to have an effect on sleep and daytime vigilance (*e.g.*, psychoactive drugs or medications, sedatives or hypnotics, including SSRI's), or (6) were left-handed.

### Experimental design (Figure 1)

In the evening between 8 and 9 PM, all subjects performed the psychomotor vigilance task (PVT) and then trained on the motor sequence task (MST). After training, participants spent the night in the laboratory and underwent standard sleep recording. The next morning between 6:30 and 7:30 AM subjects repeated the PVT and were tested on the MST. After a 10 minute break, they then learned a new MST sequence, again with 12 trials, to control for circadian effects of motor sequence learning. MST sequences were counterbalanced across subjects within groups to control for any order effect. Learning the motor sequence task is sequence specific, with no transference of learning to new sequences [12].

### Study procedures

#### PVT

The PVT measures sustained attention and reaction time and has been shown to be sensitive to sleep deprivation, partial sleep loss, and circadian variation in performance efficiency [21]. In this task, subjects push a button as fast as they can whenever they see a small (3 mm high, 4 digits wide) LED millisecond clock begin counting up from 0000. Pressing the button stops the digital clock, allowing the subject 1.5 seconds to read the reaction time (RT). The inter-stimulus interval on the task varies randomly from 2 to 10 seconds. The duration of the task can be either 5 – as in our case - or 10 minutes. It has only a 1–3 trial learning curve.

#### Motor Sequence Task (MST)

The MST requires subjects to type repeatedly a 5-element number on a standard computer keyboard with their non-dominant left hands. Subjects were asked to type either [4-1-3-2-4] or [2-4-1-3-2]. The specific sequence, which must be typed, is displayed in front of subjects on the computer screen at all times. Typing is done in 30 second trials separated by 30 second rest periods. Subjects fixate on this number while typing. Training and retest each involves 12 trials.

The main performance measure was the number of correctly typed sequences per 30-second trial, thus reflecting both speed and accuracy. The primary outcome measure was the initial overnight improvement, calculated as the percent increase in sequences from the last three training trials in the evening session to the first three in the morning session [22]. (Figure 2)

#### Polysomnography and scoring

Standard overnight PSG recording and data interpretation were performed in accordance with the American Academy of Sleep Medicine (AASM) scoring manual [23], [24]. This included standard electroencephalogram (EEG) leads (F1, F2, C3, C4, O1, and O2). In addition, bilateral electrooculogram (EOG), submental electromyogram (EMG), bilateral anterior tibialis electromyogram (EMG), and standard electrocardiogram (ECG) electrodes were employed. We also recorded nasal/oral airflow (thermistor), nasal pressure (Validyne transducer), chest plus abdominal wall motion (piezo electrodes) and oxygen saturation.

All studies were scored by a registered PSG technologist, blinded to subject performance. In particular, hypopneas required a clear (discernable) amplitude reduction of a validated measure of breathing during sleep, and were associated with either an oxygen desaturation of >3% or an arousal lasting ≥10 sec. Arousals were scored visually according to the AASM manual scoring criteria, which require an abrupt shift of EEG frequency including alpha, theta and/or frequencies greater than 16 Hz (but not spindles) that last at least 3 seconds with 10 seconds of stable sleep preceding.

### Statistical analysis

Statistical analysis was performed using JMP Version 8 (SAS Institute Inc., Cary, NC). Unpaired t-tests were performed to compare the demographic, questionnaire and PSG data between OSA patients and healthy controls. We calculated the MST percent improvement for each subject from initial evening training to subsequent morning retesting. Comparisons were made between OSA patients and controls using an unpaired t-test for MST improvement. Regression analyses were performed to separate influences of AHI, oxygen nadir, and arousal index on overnight performance changes. A *p*-value of <0.05 was considered significant. Variability is expressed as standard errors of the mean (SEM).

### Sample Size Justification

For the motor sequence task, published results indicate an effect size of 1.64. The required sample size to achieve a power of 80% with the alpha-level set to 0.05, is an n = 14 for a two-tailed test and n = 12 for a single-tailed test.
